# Supplementary material for: Astrocyte molecular rhythm disruption in nucleus accumbens promotes increased binge-like drinking in mice
Source: bioRxiv. 2026 Sep 2:2026.08.28.747847. Preprint. [Version 1] doi: 10.64898/2026.08.28.747847 (PMC13674595; doi:10.64898/2026.08.28.747847)
Supplement: Supplementary file 1 [file media-2.pdf]

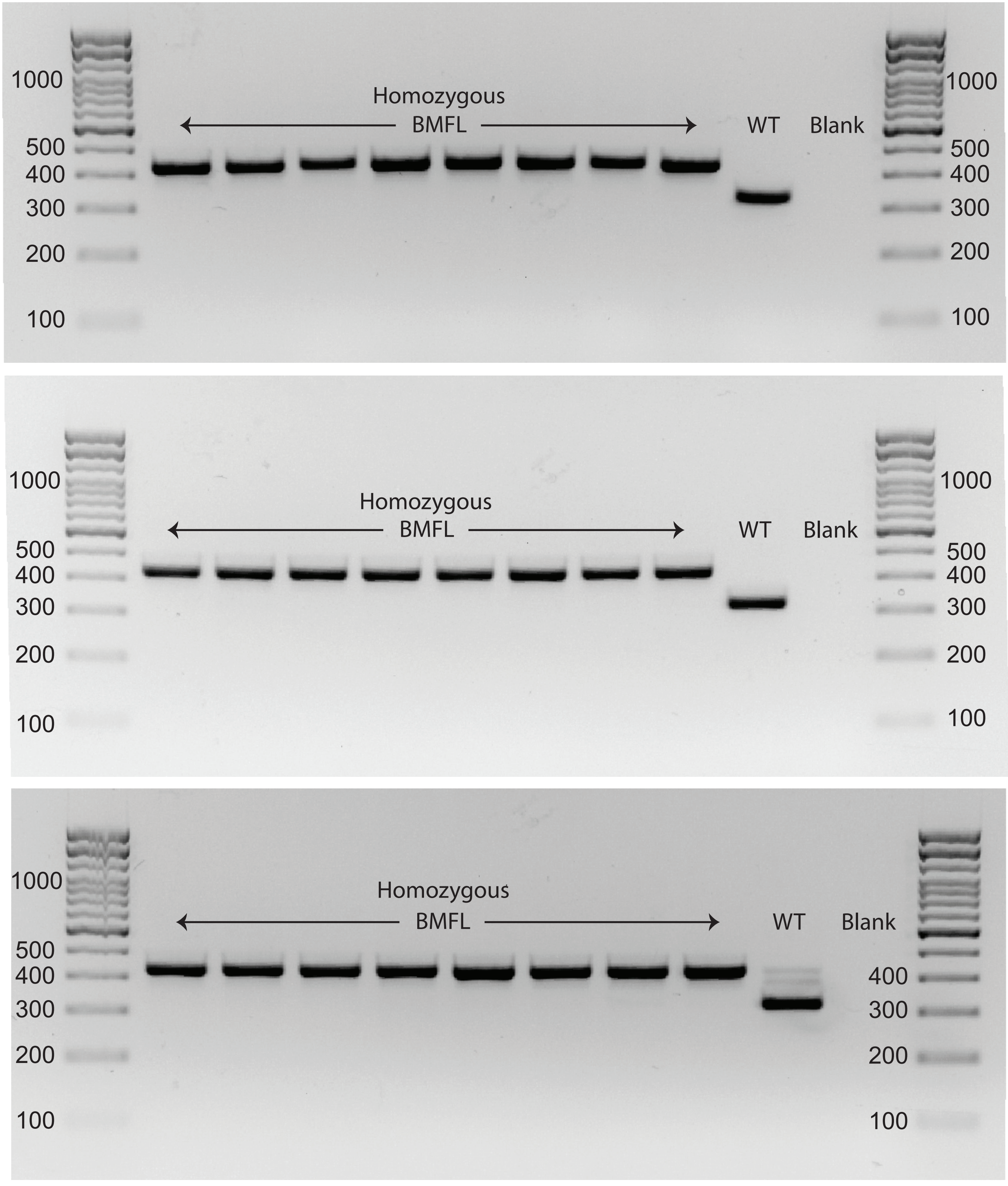
1000
Homozygous
BMFL
WT
Blank
1000
500
400
300
200
100
500
400
300
200
100
1000
Homozygous
BMFL
WT
Blank
1000
500
400
300
200
100
500
400
300
200
100
1000
Homozygous
BMFL
WT
Blank
500
400
300
200
100
400
300
200
100
