# Supplementary material for: Astrocyte molecular rhythm disruption in nucleus accumbens promotes increased binge-like drinking in mice
Source: bioRxiv. 2026 Sep 2:2026.08.28.747847. Preprint. [Version 1] doi: 10.64898/2026.08.28.747847 (PMC13674595; doi:10.64898/2026.08.28.747847)
Supplement: Supplementary file 2 [file media-3.pdf]

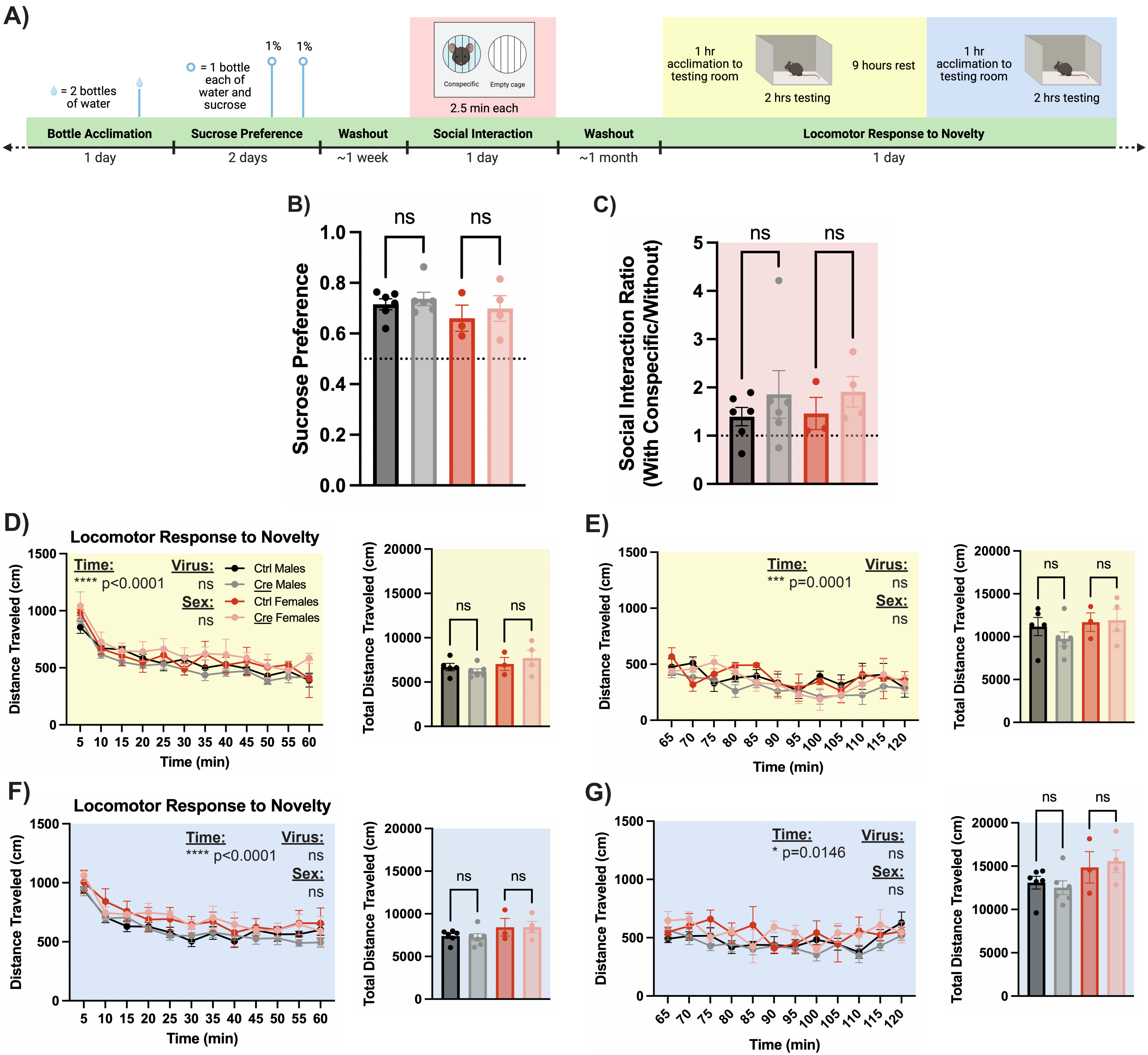
A)
= 2 bottles of water
= 1 bottle each of water and sucrose
1%
1%
Conspecific
Empty cage
2.5 min each
1 hr acclimation to testing room
2 hrs testing
9 hours rest
1 hr acclimation to testing room
2 hrs testing
Bottle Acclimation
Sucrose Preference
Washout
Social Interaction
Washout
Locomotor Response to Novelty
1 day
2 days
~1 week
1 day
~1 month
1 day
B)
Sucrose Preference
ns
ns
C)
Social Interaction Ratio (With Conspecific/Without)
ns
ns
D)
Locomotor Response to Novelty
Time:
**** p<0.0001
Virus:
ns
Sex:
ns
Ctrl Males
Cre Males
Ctrl Females
Cre Females
Distance Traveled (cm)
Time (min)
Total Distance Traveled (cm)
ns
ns
E)
Time:
*** p=0.0001
Virus:
ns
Sex:
ns
Distance Traveled (cm)
Time (min)
Total Distance Traveled (cm)
ns
ns
F)
Locomotor Response to Novelty
Time:
**** p<0.0001
Virus:
ns
Sex:
ns
Distance Traveled (cm)
Time (min)
Total Distance Traveled (cm)
ns
ns
G)
Time:
* p=0.0146
Virus:
ns
Sex:
ns
Distance Traveled (cm)
Time (min)
Total Distance Traveled (cm)
ns
ns
