# Supplementary material for: Astrocyte molecular rhythm disruption in nucleus accumbens promotes increased binge-like drinking in mice
Source: bioRxiv. 2026 Sep 2:2026.08.28.747847. Preprint. [Version 1] doi: 10.64898/2026.08.28.747847 (PMC13674595; doi:10.64898/2026.08.28.747847)
Supplement: Supplementary file 3 [file media-4.pdf]

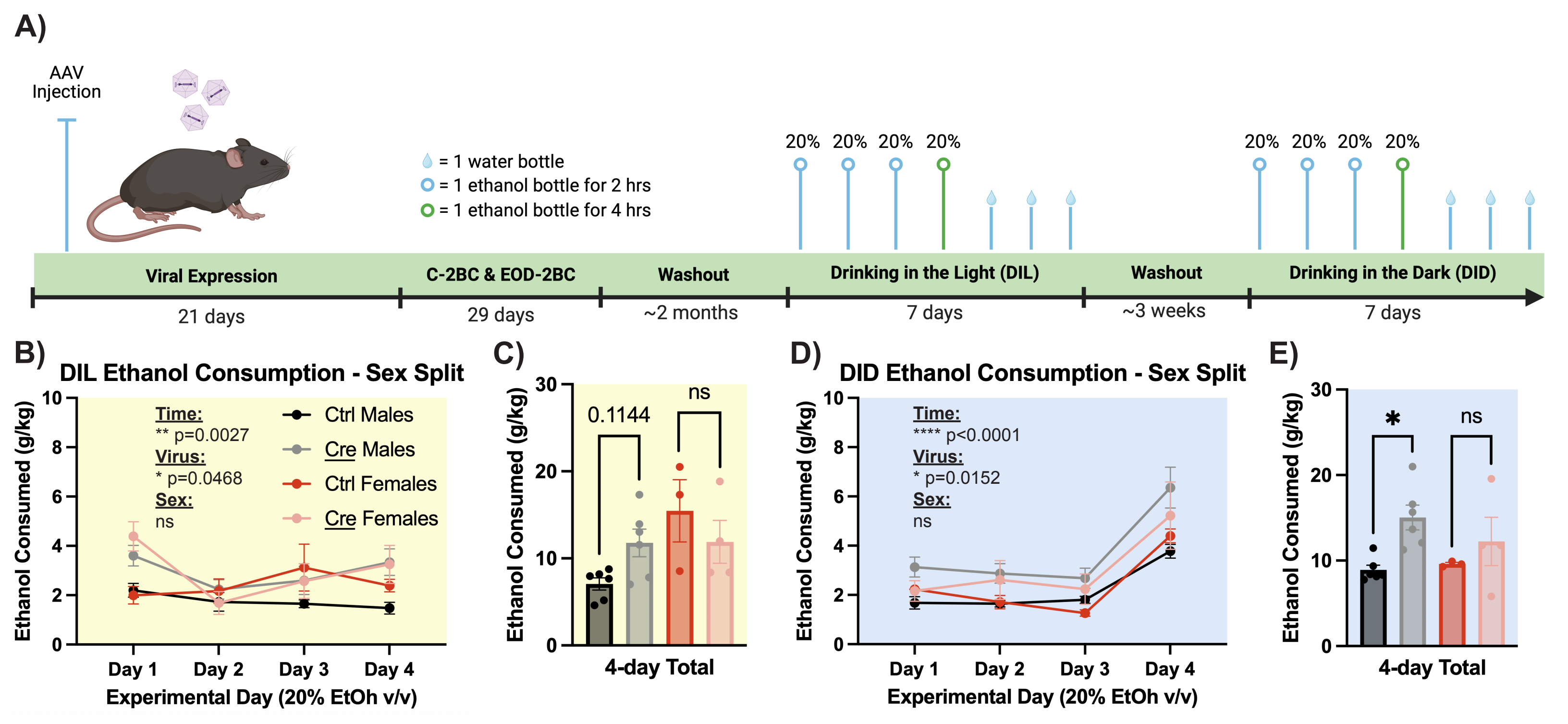
A)
AAV Injection
= 1 water bottle
= 1 ethanol bottle for 2 hrs
= 1 ethanol bottle for 4 hrs
20% 20% 20% 20%
20% 20% 20% 20%
Viral Expression
C-2BC & EOD-2BC
Washout
Drinking in the Light (DIL)
Washout
Drinking in the Dark (DID)
21 days
29 days
~2 months
7 days
~3 weeks
7 days
B)
DIL Ethanol Consumption - Sex Split
Time:
** p=0.0027
Virus:
* p=0.0468
Sex:
ns
Ctrl Males
Cre Males
Ctrl Females
Cre Females
Ethanol Consumed (g/kg)
Day 1 Day 2 Day 3 Day 4
Experimental Day (20% EtOh v/v)
C)
0.1144
ns
Ethanol Consumed (g/kg)
4-day Total
D)
DID Ethanol Consumption - Sex Split
Time:
**** p<0.0001
Virus:
* p=0.0152
Sex:
ns
Ethanol Consumed (g/kg)
Day 1 Day 2 Day 3 Day 4
Experimental Day (20% EtOh v/v)
E)
*
ns
Ethanol Consumed (g/kg)
4-day Total
